# Supplementary figures and images for: Early Embryonic Loss Following Intravaginal Zika Virus Challenge in Rhesus Macaques
Source: Front Immunol. 2021 May 17;12:686437. doi: 10.3389/fimmu.2021.686437 (PMC8165274; doi:10.3389/fimmu.2021.686437)

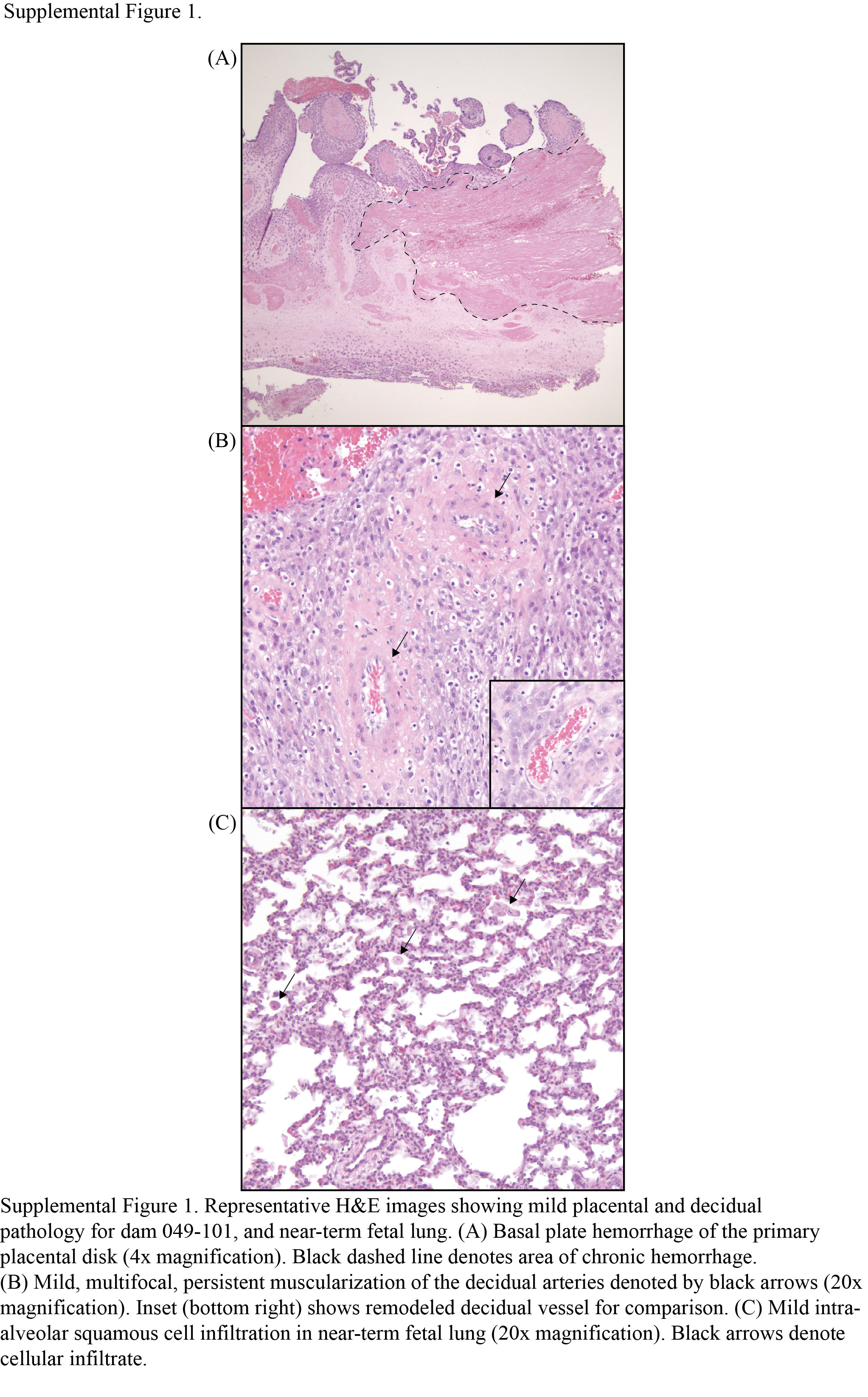

Supplement: Supplementary file 1 [file Image_1.jpeg]

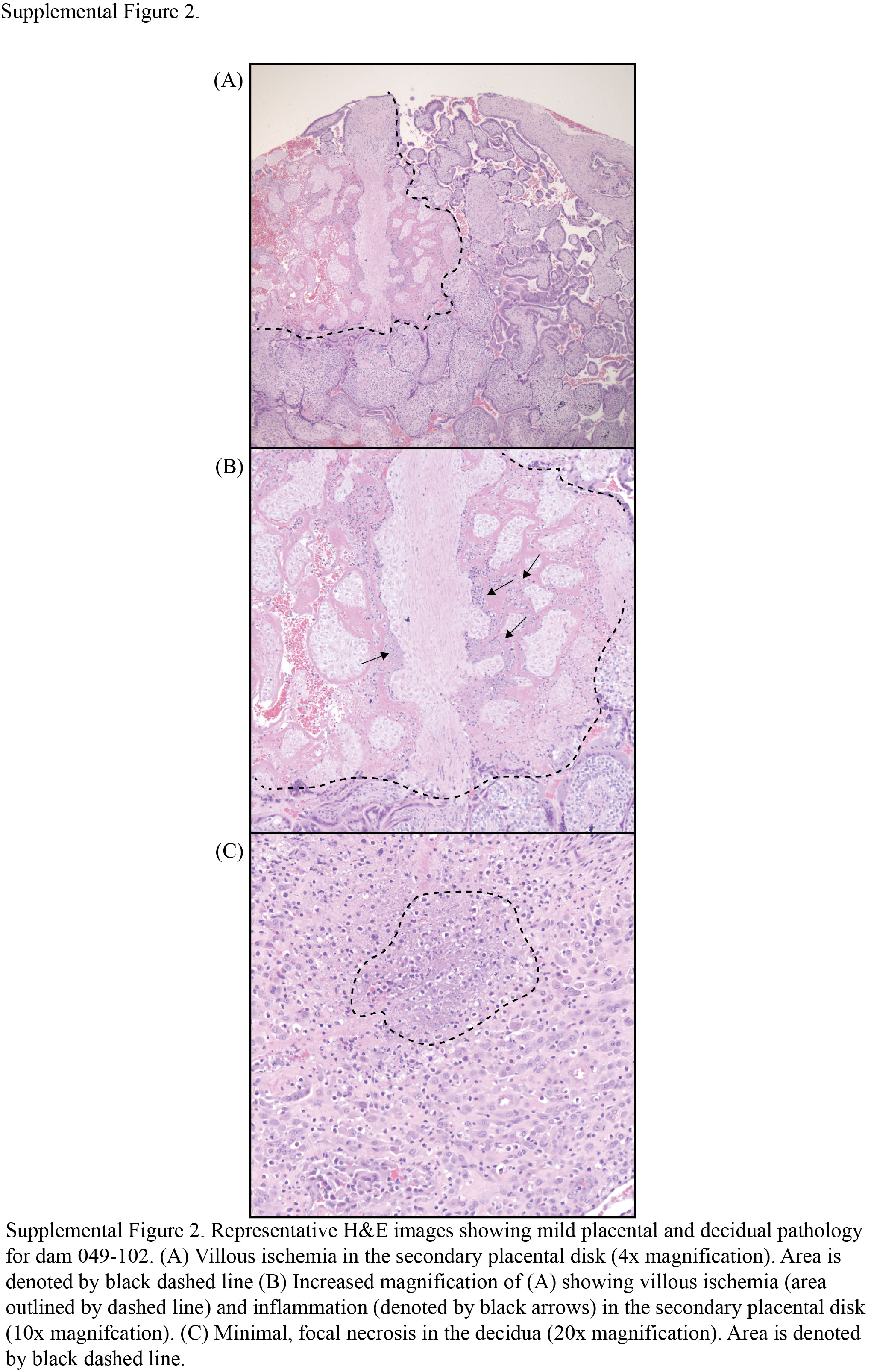

Supplement: Supplementary file 2 [file Image_2.jpeg]

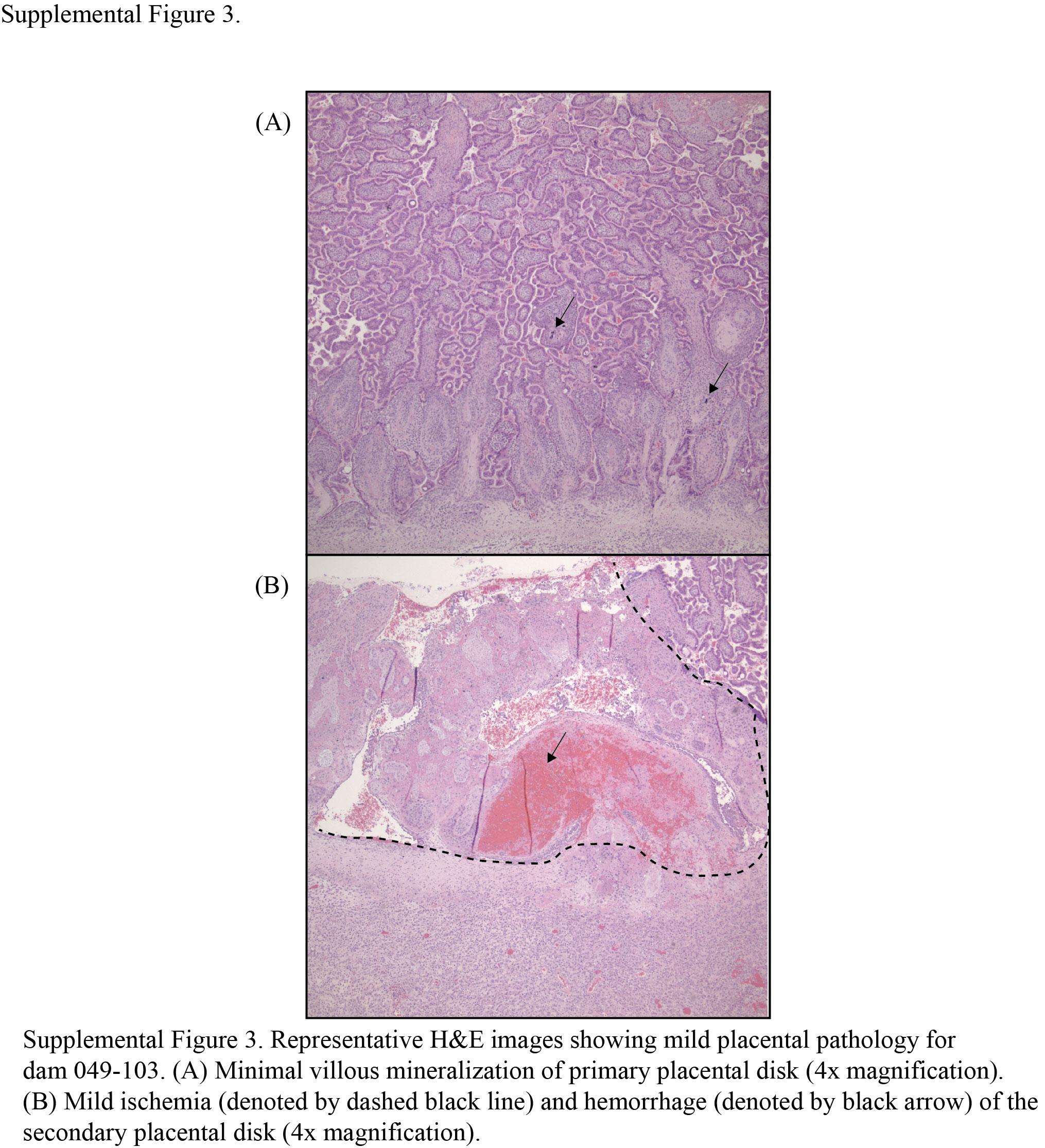

Supplement: Supplementary file 3 [file Image_3.jpeg]
